# Supplementary material for: Within-subject variation of C-reactive protein and high-sensitivity C-reactive protein: A systematic review and meta-analysis
Source: PLoS One. 2024 Nov 1;19(11):e0304961. doi: 10.1371/journal.pone.0304961 (PMC11530069; doi:10.1371/journal.pone.0304961)
Supplement: S1 File — (DOCX) [file pone.0304961.s002.docx]

# Appendix 1

Search strategies

#### (Medline, Embase, Medline and In-Process, In-Data-Review & Other non-indexed citations)

Sample search strategy, Medline 1946-August 05 2020 and In-Process and other non-indexed citations

1. (CRP or c-reactive protein).mp. [mp=title, abstract, original title, name of substance word, subject heading word, floating sub-heading word, keyword heading word, organism supplementary concept word, protocol supplementary concept word, rare disease supplementary concept word, unique identifier, synonyms]

2. Glycated Hemoglobin A/ or HbA1c.mp.

3. biological varia$.mp. [mp=title, abstract, original title, name of substance word, subject heading word, floating sub-heading word, keyword heading word, organism supplementary concept word, protocol supplementary concept word, rare disease supplementary concept word, unique identifier, synonyms]

4. within-individual varia$.mp.

5. within-subject varia$.mp. [mp=title, abstract, original title, name of substance word, subject heading word, floating sub-heading word, keyword heading word, organism supplementary concept word, protocol supplementary concept word, rare disease supplementary concept word, unique identifier, synonyms]

6. within-individual CV.mp. [mp=title, abstract, original title, name of substance word, subject heading word, floating sub-heading word, keyword heading word, organism supplementary concept word, protocol supplementary concept word, rare disease supplementary concept word, unique identifier, synonyms]

7. Within-subject CV.mp. [mp=title, abstract, original title, name of substance word, subject heading word, floating sub-heading word, keyword heading word, organism supplementary concept word, protocol supplementary concept word, rare disease supplementary concept word, unique identifier, synonyms]

8. Intraindividual CV.mp. [mp=title, abstract, original title, name of substance word, subject heading word, floating sub-heading word, keyword heading word, organism supplementary concept word, protocol supplementary concept word, rare disease supplementary concept word, unique identifier, synonyms]

9. intra-individual varia$.mp.

10. day-to-day.mp.

11. visit-to-visit.mp.

12. Coefficient of variation.mp.

13. (cvi or cvw or cvb or cvwithin-subject or cvbiological).mp. [mp=title, abstract, original title, name of substance word, subject heading word, floating sub-heading word, keyword heading word, organism supplementary concept word, protocol supplementary concept word, rare disease supplementary concept word, unique identifier, synonyms]

14. chronic venous insufficiency.mp.

15. vaccine immunology.mp.

16. content validity index.mp.

17. cerebral visual impairment.mp.

18. community virtual ward.mp.

19. cardiac vagal withdrawal.mp.

20. coronary vessel wall.mp.

21. variability independent of the mean.mp.

22. VIM.mp.

23. thalamus.mp. or Thalamus/

24. lactamase.mp. or beta-Lactamases/ or beta-Lactamase Inhibitors/

25. index of individuality.mp.

26. Reference Change Value.mp.

27. rcv.mp.

28. (intraclass or ICC).mp. [mp=title, abstract, original title, name of substance word, subject heading word, floating sub-heading word, keyword heading word, organism supplementary concept word, protocol supplementary concept word, rare disease supplementary concept word, unique identifier, synonyms]

29. Revue canadienne du vieillissement.mp.

30. recoverin.mp. or Recoverin/

31. index of heterogeneity.mp.

32. validity coefficient.mp.

33. reliability parameter.mp.

34. (animal not human).mp. [mp=title, abstract, original title, name of substance word, subject heading word, floating sub-heading word, keyword heading word, organism supplementary concept word, protocol supplementary concept word, rare disease supplementary concept word, unique identifier, synonyms]

35. 1 or 2

36. 3 or 4 or 5 or 6 or 7 or 8 or 9 or 10 or 11 or 12 or 13 or 25 or 26 or 28 or 31 or 32 or 33

37. 14 or 15 or 16 or 17 or 18 or 19 or 20 or 23 or 24 or 29 or 30

38. 35 and 36

39. 38 not 37

40. 39 not 34

## Appendix 2

Table 4 Databases searched with dates of search coverage

| **Database**  *Ovid*  Medline  Embase  Medline and In-Process, In-Data-Review & Other non-indexed citations  Cochrane Central  Epistemonikos  Open Grey | **Coverage**  1946 to 11/22  1974 to 11/22  1946 to 11/22  Inception to 11/22  Inception to 11/22  Inception to 11/22 |
| --- | --- |

# Appendix 3

## COSMIN Risk of bias template

| Element | Instruction | Result |
| --- | --- | --- |
| 1. Name of the instrument | CRP, hsCRP or HbA1c |  |
| 2. Version or way of operationalization | Method of analysis |  |
| 3. Construct | Description of what is being measured |  |
| 4. Measurement property. | Variability measure(s) |  |
| 5. Components that will be repeated | Whole measurement? Parts? | Whole |
| 6. Source(s) of variation varied | Component which is varied across the measurements (time?) Unintentional sources of variation | Time |
| 7. Patient population |  |  |
|  |  |  |

Part A: Elements of Research Question

Notes:

4. Reliability study (relative eg ICC) or measurement error eg SD, CV, and which measurement

6. Any deficiencies that may introduce unintentional sources of variation eg training, preparation, collection, transport, test methodology

Part B: Risk of bias

| Design requirements | Rating* | Reason/evidence |
| --- | --- | --- |
| 1. Were patients stable in the time between the repeated measurements on the construct to be measured? |  |  |
| 2. Was the time interval between the repeated measurements appropriate? |  |  |
| 3. Were the measurement conditions similar for the repeated measurements – except for the condition being evaluated as a source of variation? |  |  |
| 4. Did the professional(s) administer the measurement without knowledge of scores or values of other repeated measurement(s) in the same patients? |  |  |
| 5. Did the professional(s) assign scores or determine values without knowledge of the scores or values of other repeated measurement(s) in the same patients? |  |  |
| 6. Were there any other important flaws in the design or statistical methods of the study? |  |  |
| 7. Was the Coefficient of Variation (CV), ICC, RCV, SD, VIM, variance, ASV, II calculated? |  |  |
|  |  |  |

* Very good/adequate/doubtful/inadequate/NA

Paper notes:

Guidance Notes:

Ratings are as follows unless described otherwise below:

Very good: yes; Adequate: reasons to assume standards were met; Doubtful: unclear; Inadequate (no)

1. Completely stable is very good, long term illness is adequate, short term/acute illness is doubtful, unstable should have been excluded at screening

2. Any time interval is allowed for the purposes of this review

3. Environmental change may count as doubtful

6. Very good: no; Doubtful: minor methodological flaws; inadequate: yes

7. Very good: the model or formula for the variability measure is described; it matches the reviewer constructed research question and the data;

Adequate: the model or formula is not described or does not optimally match the reviewer constructed research question and evidence provided that no systematic difference has occurred

Doubtful: no knowledge about systematic difference or with evidence provided that systematic difference has occurred

Inadequate: variability calculated based on Cronbach’s alpha, or using SD from another population

# Appendix 4

## Statistical calculations for ICC meta-analysis

Z = 0.5 * ln((1 + ICC) / (1 - ICC)). Standard error was calculated using the formula SE = sqrt(1/(N - 3) Z scores were then back-transformed

## Stata code

### For ICC:

gen z = 0.5 * ln((1 + PrimarymeasureofICCseentoe ) / (1 - PrimarymeasureofICCseentoe ))

gen sez = sqrt(1/( Totalsubjectsinprimaryanalys - 3))

meta set z sez, random(dl) studylabel( Shorttitle ) studysize( Totalsubjectsinprimaryanalys )

meta forestplot

gen iccbk = (exp(2*z) - 1)/(exp(2*z) + 1)

gen cil = (exp(2* _meta_cil ) - 1)/(exp(2* _meta_cil ) + 1)

gen ciu = (exp(2* _meta_ciu ) - 1)/(exp(2* _meta_ciu ) + 1)

metan iccbk cil ciu if CRPhsCRP==1 , by ( Healthstatusname ) random label( namevar=Shorttitle) rcols ( Totalsubjectsinprimaryanalys Averagenumberofmeasurementso ) xlabel (0, 0.2, 0.4, 0.6, 0.8, 1.0) xtitle ("ICC")

metan iccbk cil ciu if CRPhsCRP == 1, random by ( Settingcodetext ) label ( namevar=Shorttitle) xlabel (0, 0.2, 0.4, 0.6, 0.8, 1.0) rcols ( Totalsubjectsinprimaryanalys ) xtitle (ICC)

metan iccbk cil ciu if CRPhsCRP == 2, random by ( Settingcodetext ) label ( namevar=Shorttitle) xlabel (0, 0.2, 0.4, 0.6, 0.8, 1.0) rcols ( Totalsubjectsinprimaryanalys ) xtitle (ICC)

metan iccbk cil ciu if CRPhsCRP == 1, random by ( Healthstatusname ) label ( namevar=Shorttitle) xlabel (0, 0.2, 0.4, 0.6, 0.8, 1.0) rcols ( Totalsubjectsinprimaryanalys ) xtitle (ICC)

metan iccbk cil ciu if CRPhsCRP == 2, random by ( Healthstatusname ) label ( namevar=Shorttitle) xlabel (0, 0.2, 0.4, 0.6, 0.8, 1.0) rcols ( Totalsubjectsinprimaryanalys ) xtitle (ICC)

metan iccbk cil ciu if ( Totalsubjectsinprimaryanalys >100), random by ( HbA1cCRPhsCRPormultiple ) label ( namevar=Shorttitle) xlabel (0, 0.2, 0.4, 0.6, 0.8, 1.0) rcols ( Totalsubjectsinprimaryanalys ) xtitle (ICC)

metan iccbk cil ciu if ( ROBHighest <3), random by ( HbA1cCRPhsCRPormultiple ) label ( namevar=Shorttitle) xlabel (0, 0.2, 0.4, 0.6, 0.8, 1.0) rcols ( Totalsubjectsinprimaryanalys ) xtitle (ICC)

metan iccbk cil ciu if ( CRPhsCRP ==1 & Shortorlongtermvariability == "L" ), random label ( namevar=Shorttitle) xlabel (0, 0.2, 0.4, 0.6, 0.8, 1.0) rcols ( Totalsubjectsinprimaryanalys ) xtitle (ICC)

metan iccbk cil ciu if ( CRPhsCRP ==2 & Shortorlongtermvariability == "L" ), random label ( namevar=Shorttitle) xlabel (0, 0.2, 0.4, 0.6, 0.8, 1.0) rcols ( Totalsubjectsinprimaryanalys ) xtitle (ICC)

metan iccbk cil ciu if ( CRPhsCRP ==1 ), random by ( Unitofmeasurementofmeasurand ) label ( namevar=Shorttitle) xlabel (0, 0.2, 0.4, 0.6, 0.8, 1.0) rcols ( Totalsubjectsinprimaryanalys ) xtitle (ICC)

metan iccbk cil ciu if ( CRPhsCRP ==2 ), random by ( Unitofmeasurementofmeasurand ) label ( namevar=Shorttitle) xlabel (0, 0.2, 0.4, 0.6, 0.8, 1.0) rcols ( Totalsubjectsinprimaryanalys ) xtitle (ICC)

# Appendix 5

Table 5 Study characteristics detail

| Paper | CRP or hSCRP | Number of subjects | Age (primary analysis) | % male (primary analysis) |
| --- | --- | --- | --- | --- |
| Alexander 2013 [25] | hsCRP | 15 |  | 40.00 |
| Bailey 2014 [26] | CRP | 27 |  | 45.00 |
| Bay-Jensen 2017 [27] | hsCRP | 490 |  |  |
| Becker 2020 [28] | hsCRP | 11 | 18 | 100 |
| Block 2006 [29] | CRP | 206 | 45.4 |  |
| Blum 2005 [30] | hsCRP | 15 |  | 0.00 |
| Boenisch 2002 [31] | CRP | 10 | 67 | 10.00 |
| Bogaty 2013 [32] | hsCRP | 100 | 65.6 | 100.00 |
| Bogaty 2005 [33] | hsCRP | 159 |  |  |
| Browning 2004 [34] | CRP | 15 |  | 0.00 |
| Carobene 2019 [35] | CRP | 87 |  | 42.53 |
| Cho 2005 [36] | CRP | 11 | 30 | 0.00 |
| Chrismas 2018 [37] | hsCRP | 50 | 26 | 72.00 |
| Clark 1993 [38] | CRP | 19 |  | 0.47 |
| Corte 2020 [39] | CRP | 11 |  | 0.27 |
| De Maat 1996 [40] | CRP | 20 | 31 |  |
| DeGoma 2012 [41] | CRP | 255 | 59 | 60.00 |
| D'Eril 2001 [42] | CRP | 22 |  | 50.00 |
| Engelberger 2015 [43] | hsCRP | 80 | 57 | 55.00 |
| Franzini 1993 [44] | CRP | 10 |  |  |
| Gasco 2010 [45] | CRP | 16 | 52.7 | 50.00 |
| Glynn 2009 [46] | hsCRP | 8901 |  | 62.09 |
| Guy 2017 [47] | CRP | 12 | 24.3 | 100.00 |
| Hardikar 2014 [48] | hsCRP | 329 | 61 | 82.20 |
| Hosogaya 1999 [49] | CRP | 22 |  | 0.73 |
| Itoh 2012 [50] | hsCRP | 4 |  | 100.00 |
| Jackson 2015 [51] | CRP | 3410 | 65 | 44.10 |
| Jain 2020 [52] | hsCRP | 37 | 22.6 | 45.21 |
| Jung 2019 [53] | CRP | 20 |  |  |
| Kelly 2019 [24] | CRP | 90 | 52.8 | 31.00 |
| Koenig 2003 [54] | CRP | 696 |  | 100.00 |
| Kolsum 2009 [55] | CRP | 58 | 63.78 | 68.97 |
| Laclair 2008 [56] | hsCRP | 32 | 50 | 100.00 |
| Lee 2007 [57] | hsCRP | 48 | 54.8 | 10.00 |
| Macy 1997 [58] | CRP | 26 |  | 38.46 |
| Malaponte 2007 [59] | CRP | 18 | 72.86 | 55.81 |
| Meyer 2011 [60] | CRP | 467 | 24.9 | 100.00 |
| Nasermoaddeli 2006 [61] | hsCRP | 1679 |  | 0.54 |
| Navarro 2012 [62] | hsCRP | 62 | 30.4 | 50.00 |
| Nunes 2010 [63] | CRP | 56 | 18 | 100.00 |
| Ockene 2001 [64] | hsCRP | 113 | 49 | 56.64 |
| Park 2022 [65] | CRP | 89 | 70.5 | 54 |
| Platz 2010 [66] | hsCRP | 50 | 64.9 | 100.00 |
| Qi 2016 [67] | hsCRP | 40 | 46 | 52.50 |
| Riese 2002 [68] | CRP | 224 | 45.2 | 41.07 |
| Rubin 2018 [69] | CRP | 130 | 43.9 | 0.00 |
| Rudez 2009 [70] | CRP | 40 | 41 | 35.00 |
| Rutter 2022 [71] | CRP | 16549 |  |  |
| Sakkinen 1999 [72] | CRP | 26 | 27 | 38.46 |
| Sennels 2007 [73] | hsCRP | 38 | 43 | 61.00 |
| Shaw 2014 [74] | CRP | NR |  |  |
| Sjoberg 2014 [75] | CRP | 188 | 66 | 5.00 |
| Thyagarajan 2016 [76] | hsCRP | 50 |  | 63.27 |
| Thompson 2022 [77] | CRP | 272 | 62.7 | 37.8 |
| Todd 2013 [78] | hsCRP | 25 | 36 | 82.00 |
| Tsirpanlis 2004 [79] | hsCRP | 29 | 63.96 | 51.72 |
| van den Berg 2020 [80] | hsCRP | 98 | 62.8 | 77.00 |
| Waschki 2010 [81] | hsCRP | 136 | 64 |  |
| White 2017 [82] | CRP | 249 | 29.8 | 46.00 |
| Wu 2012 [83] | hsCRP | 56218 | 51.8 | 78.52 |

Table 5 (continued) Study characteristics detail

Characteristics of papers included. Setting code: 1=primary or community;2=tertiary/secondary/laboratory. Health status code: 1=Healthy; 2=depression 3=other mental health condition 4=physical health condition 5=mixed. Variability measure refers to whether the total variability (CV_I_ + CV_A_), T, or the individual variability (CV_I_), I, was reported, or whether it was uncertain, U.

| Paper | Setting code | Health status code | Average number of measurements (primary group) | Variability measure |
| --- | --- | --- | --- | --- |
| Alexander 2013 | 2 | 1 | 18.00 | I |
| Bailey 2014 | 1 | 1 | 4.00 | I |
| Bay-Jensen 2017 |  | 2 | 2.00 | T |
| Becker 2020 | 2 | 1 | 18.00 | T |
| Block 2006 | 2 | 1 | 2.00 | T |
| Blum 2005 |  | 1 | 7.00 | T |
| Boenisch 2002 |  | 4 | 12.00 |  |
| Bogaty 2013 | 2 | 3 | 15.00 | T |
| Bogaty 2005 | 2 | 3 | 3.00 | T |
| Browning 2004 | 2 | 1 | 3.00 | T |
| Carobene 2019 | 2 | 1 | 10.00 | I |
| Cho 2005 |  | 5 | 10.00 | I |
| Chrismas 2018 | 2 | 1 | 2.00 | T |
| Clark 1993 | 2 | 1 | 10.00 | I |
| Corte 2020 | 2 | 1 | 5.00 | T |
| De Maat 1996 | 2 | 1 | 9.00 | I |
| DeGoma 2012 | 1 | 1 | 2.00 | T |
| D'Eril 2001 | 2 | 1 | 5.00 | I |
| Engelberger 2015 | 2 | 1 | 4.00 | T |
| Franzini 1993 | 2 | 1 | 8.00 | I |
| Gasco 2010 |  | 4 | 12.00 | I |
| Glynn 2009 |  | 1 | 6.00 | T |
| Guy 2017 | 2 | 1 | 2.00 | T |
| Hardikar 2014 | 2 | 5 | 2.00 | T |
| Hosogaya 1999 | 2 | 1 | 12.00 | U |
| Itoh 2012 | 2 | 1 | 6.00 | T |
| Jackson 2015 | 1 | 1 | 2.00 | T |
| Jain 2020 | 2 | 2 | 2.00 | T |
| Jung 2019 | 2 | 2 | 3.00 | T |
| Kelly 2019 | 1 | 1 | 2.00 | T |
| Koenig 2003 | 1 | 1 | 2.00 | I |
| Kolsum 2009 | 1 | 2 | 2.00 | T |
| Laclair 2008 | 2 | 4 | 4.00 | I |
| Lee 2007 | 1 | 1 | 4.00 | T |
| Macy 1997 | 2 | 1 | 8.00 | I |
| Malaponte 2007 | 2 | 4 | 8.00 | T |
| Meyer 2011 | 2 | 1 | 4.00 | T |
| Nasermoaddeli 2006 | 1 | 1 | 2.00 | T |
| Navarro 2012 | 2 | 1 | 4.00 | T |
| Nunes 2010 | 2 | 1 | 4.00 | I |
| Ockene 2001 | 2 | 1 | 5.00 | T |
| Platz 2010 | 1 | 1 | 3.00 | T |
| Park 2022 | 2 | 2 | 2.00 | T |
| Qi 2016 | 2 | 1 | 5.00 | I |
| Riese 2002 | 1 | 1 | 2.50 | I |
| Rubin 2018 | 1 | 1 | 2 .5 | U |
| Rudez 2009 | 2 | 1 | 13.00 | I |
| Rutter 2022 | 2 | 1 | 2.00 | T |
| Sakkinen 1999 | 2 | 1 | 9.00 | I |
| Sennels 2007 | 1 | 1 | 6.00 | I |
| Shaw 2014 |  | 2 |  | T |
| Sjoberg 2014 | 2 | 4 | 2.00 | T |
| Thompson 2022 | 2 | 5 | 3.00 | T |
| Thyagarajan 2016 | 1 | 1 | 2.00 | I |
| Todd 2013 | 2 | 1 | 6.00 | I |
| Tsirpanlis 2004 | 2 | 4 | 16.00 | I |
| van den Berg 2020 | 2 | 3 | 4.55 | I |
| Waschki 2010 | 2 | 2 | 2.00 | U |
| White 2017 | 2 | 2 | 2.50 | T |
| Wu 2012 | 1 | 1 | 2.00 | T |

# Appendix 6

Risk of bias scores

Table 6 Maximum risk of bias score for each study with questions B4 and B5 excluded, and lowest BIVAC score (for studies that report a CV)

| Paper | COSMIN Risk of bias score | BIVAC risk of bias score |
| --- | --- | --- |
| Alexander 2013 | 1 | C |
| Bailey 2014 | 1 | C |
| Bay-Jensen 2017 | 1 | C |
| Becker 2020 | 1 |  |
| Block 2006 | 1 |  |
| Blum 2005 | 3 |  |
| Boenisch 2002 | 1 | C |
| Bogaty 2013 | 1 |  |
| Bogaty 2005 | 2 |  |
| Browning 2004 | 1 |  |
| Carobene 2019 | 1 | A |
| Cho 2005 | 1 |  |
| Chrismas 2018 | 1 | C |
| Clark 1993 | 1 | C |
| Corte 2020 | 1 | A |
| De Maat 1996 | 2 |  |
| DeGoma 2012 | 1 |  |
| D'Eril 2001 | 1 | C |
| Engelberger 2015 | 1 | C |
| Franzini 1993 | 1 | C |
| Gasco 2010 | 2 | C |
| Glynn 2009 | 1 |  |
| Guy 2017 | 1 | C |
| Hardikar 2014 | 1 |  |
| Hosogaya 1999 | 1 | C |
| Itoh 2012 | 1 | C |
| Jackson 2015 | 2 |  |
| Jain 2020 | 1 |  |
| Jung 2019 | 3 | D |
| Kelly 2019 | 2 |  |
| Koenig 2003 | 1 |  |
| Kolsum 2009 | 3 |  |
| Laclair 2008 | 1 |  |
| Lee 2007 | 2 |  |
| Macy 1997 | 1 | C |
| Malaponte 2007 | 1 | C |
| Meyer 2011 | 2 | C |
| Nasermoaddeli 2006 | 2 |  |
| Navarro 2012 | 1 |  |
| Nunes 2010 | 1 | C |
| Ockene 2001 | 1 |  |
| Park 2022 | 2 |  |
| Platz 2010 | 1 |  |
| Qi 2016 | 1 | C |
| Riese 2002 | 2 | C |
| Rubin 2018 | 3 |  |
| Rudez 2009 | 1 | C |
| Rutter 2022 | 1 |  |
| Sakkinen 1999 | 1 | C |
| Sennels 2007 | 1 | C |
| Shaw 2014 | 3 |  |
| Sjoberg 2014 | 2 |  |
| Thompson 2022 | 2 |  |
| Thyagarajan 2016 | 2 | C |
| Todd 2013 | 1 | C |
| Tsirpanlis 2004 | 2 |  |
| van den Berg 2020 | 1 | C |
| Waschki 2010 | 3 |  |
| White 2017 | 3 | C |
| Wu 2012 | 2 |  |

# Appendix 7

Forest plots of subgroup analyses

### CRP CVs


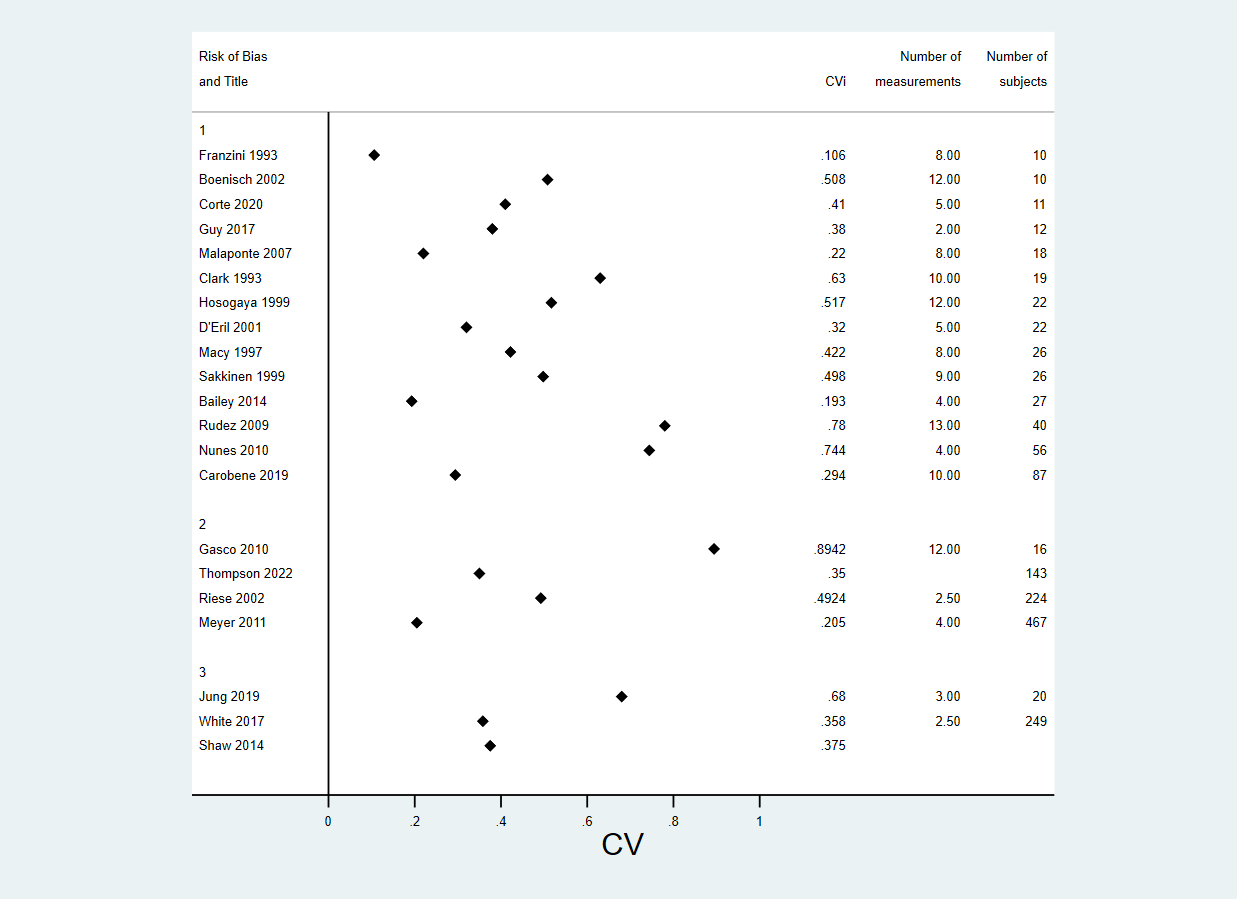


Fig 6 Forest plot of all papers that reported a CV for CRP, split by Risk of Bias


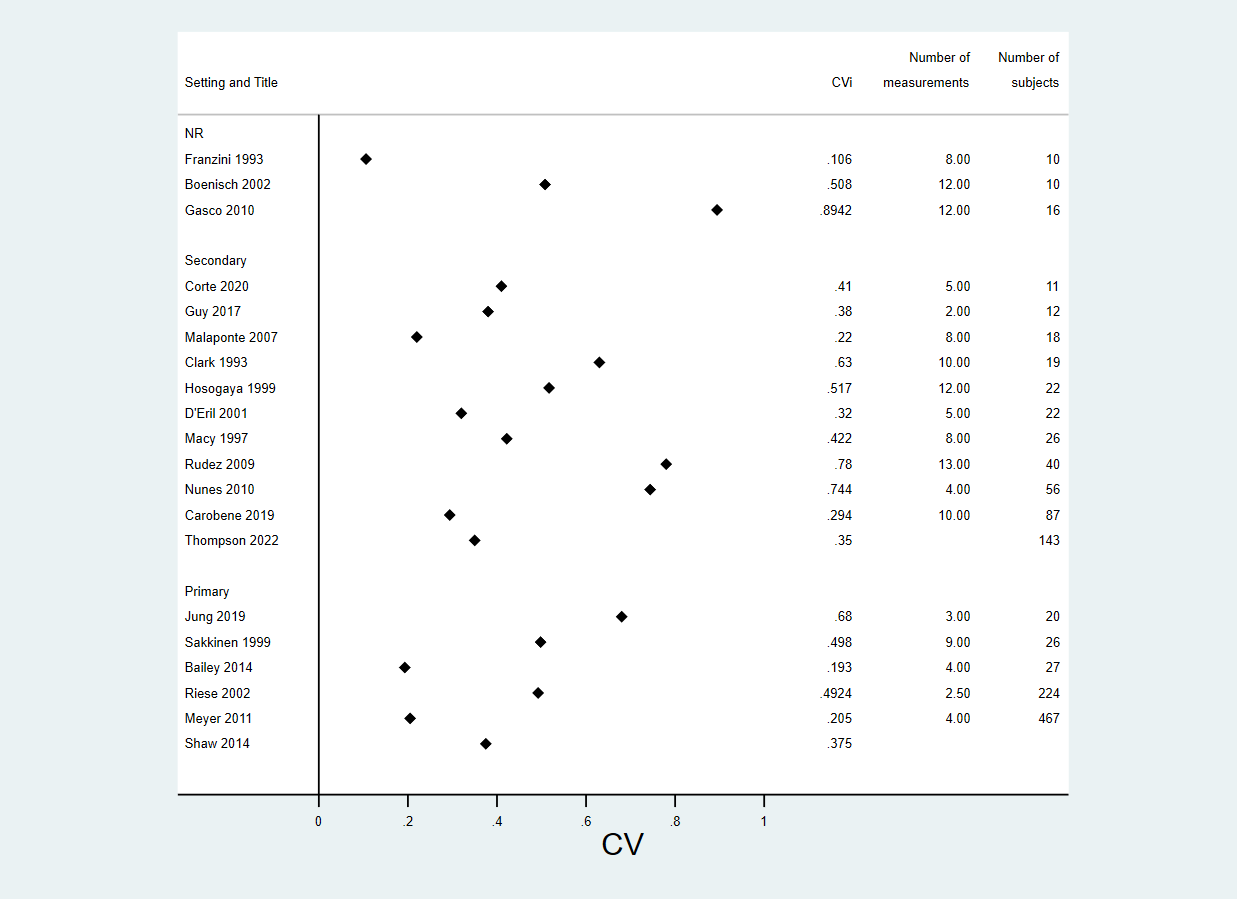


Fig 7 Forest plot of all papers that reported a CV for CRP, split by setting


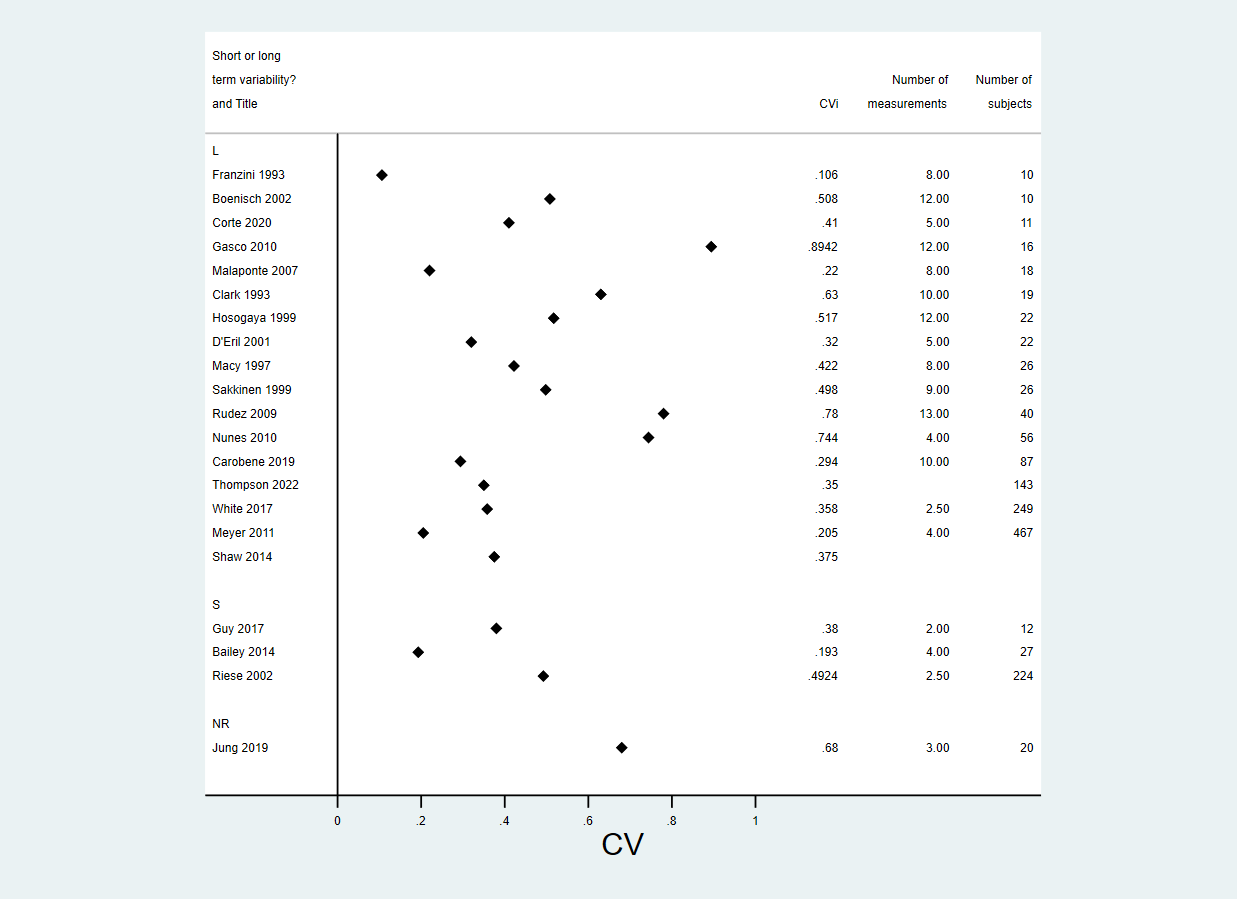


Fig 8 Forest plot of all papers that reported a CV for CRP, split by long (L) or short (S) term measurement of variability


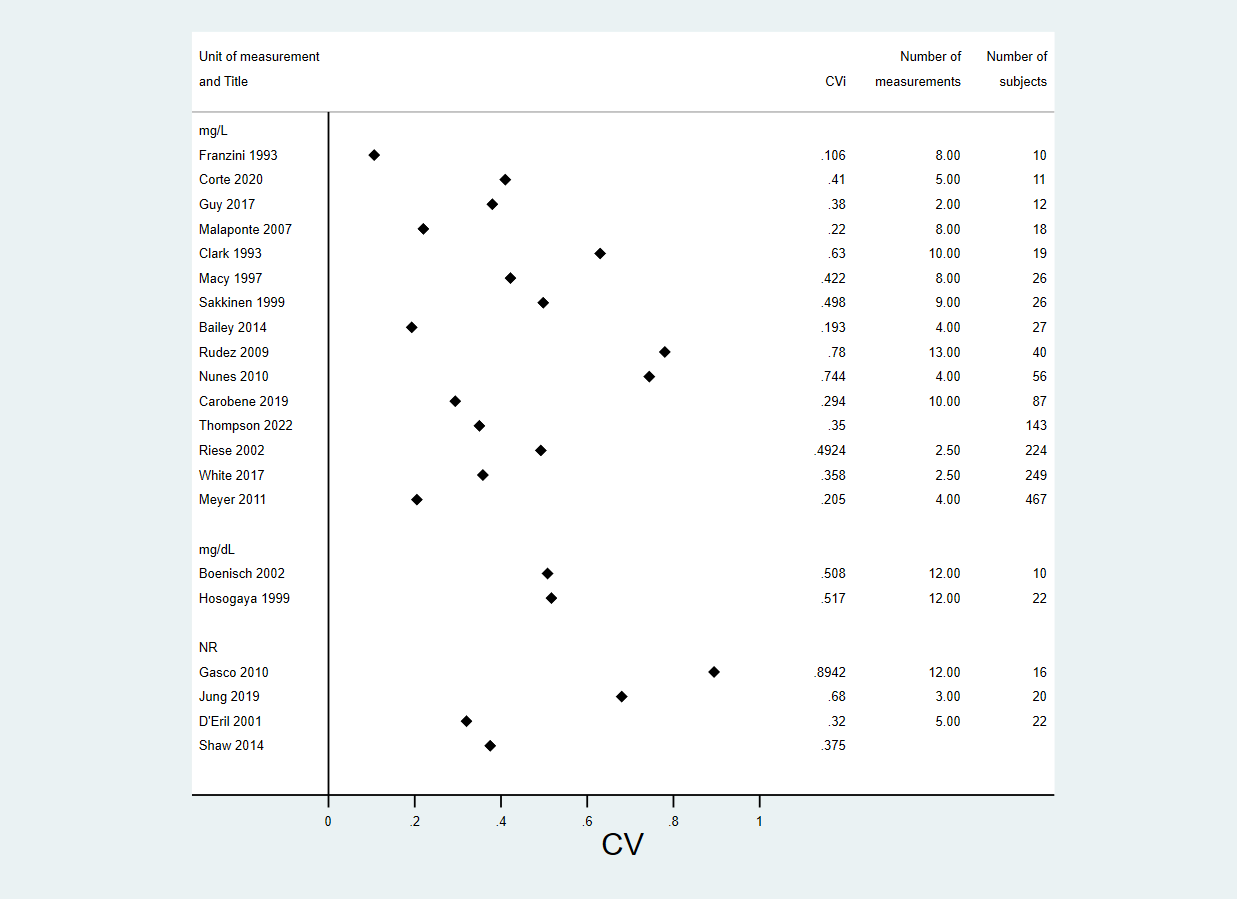


Fig 9 Forest plot of all papers that reported a CV for CRP, split by unit of measurement

### hsCRP CVs


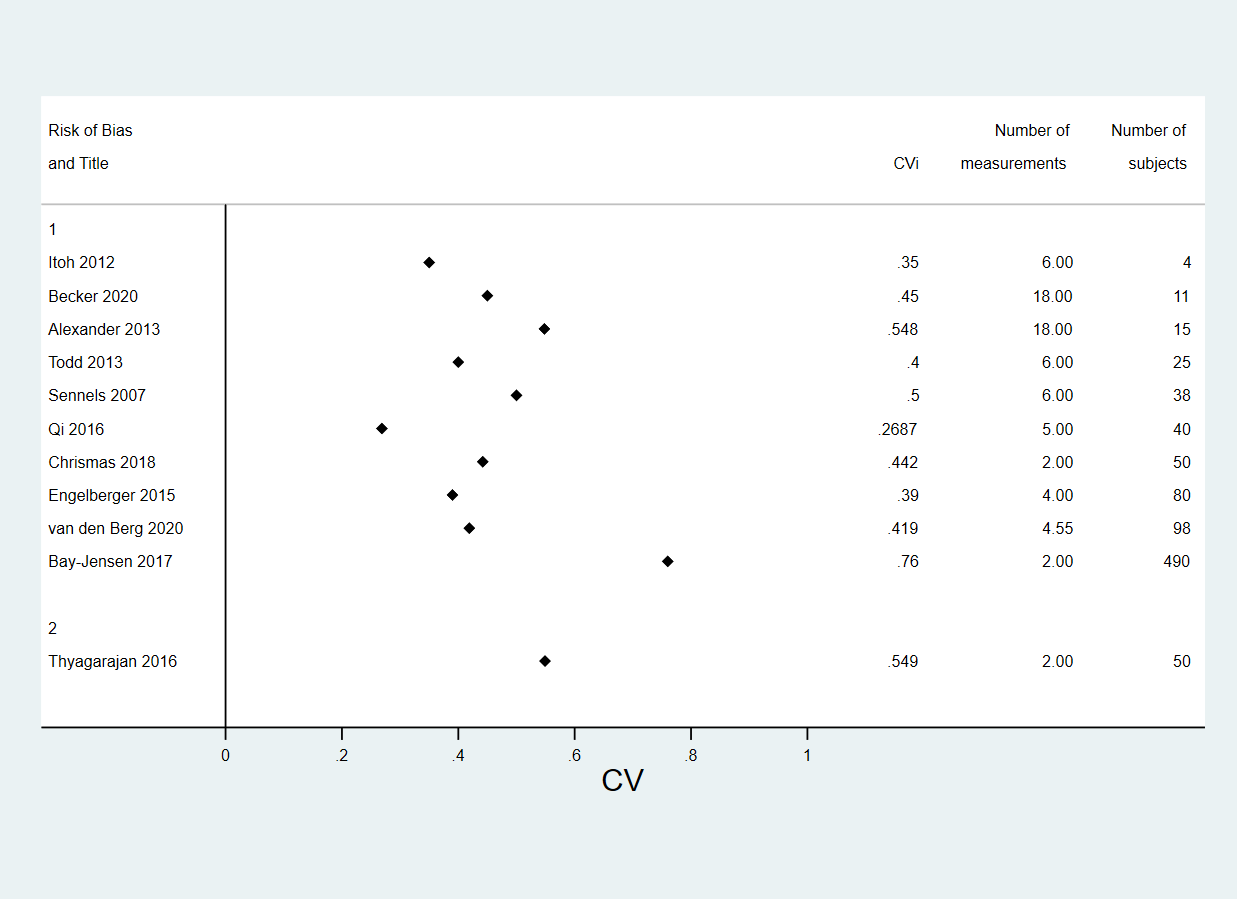


Fig 10 Forest plot of all papers that reported a CV for hsCRP, split by Risk of Bias


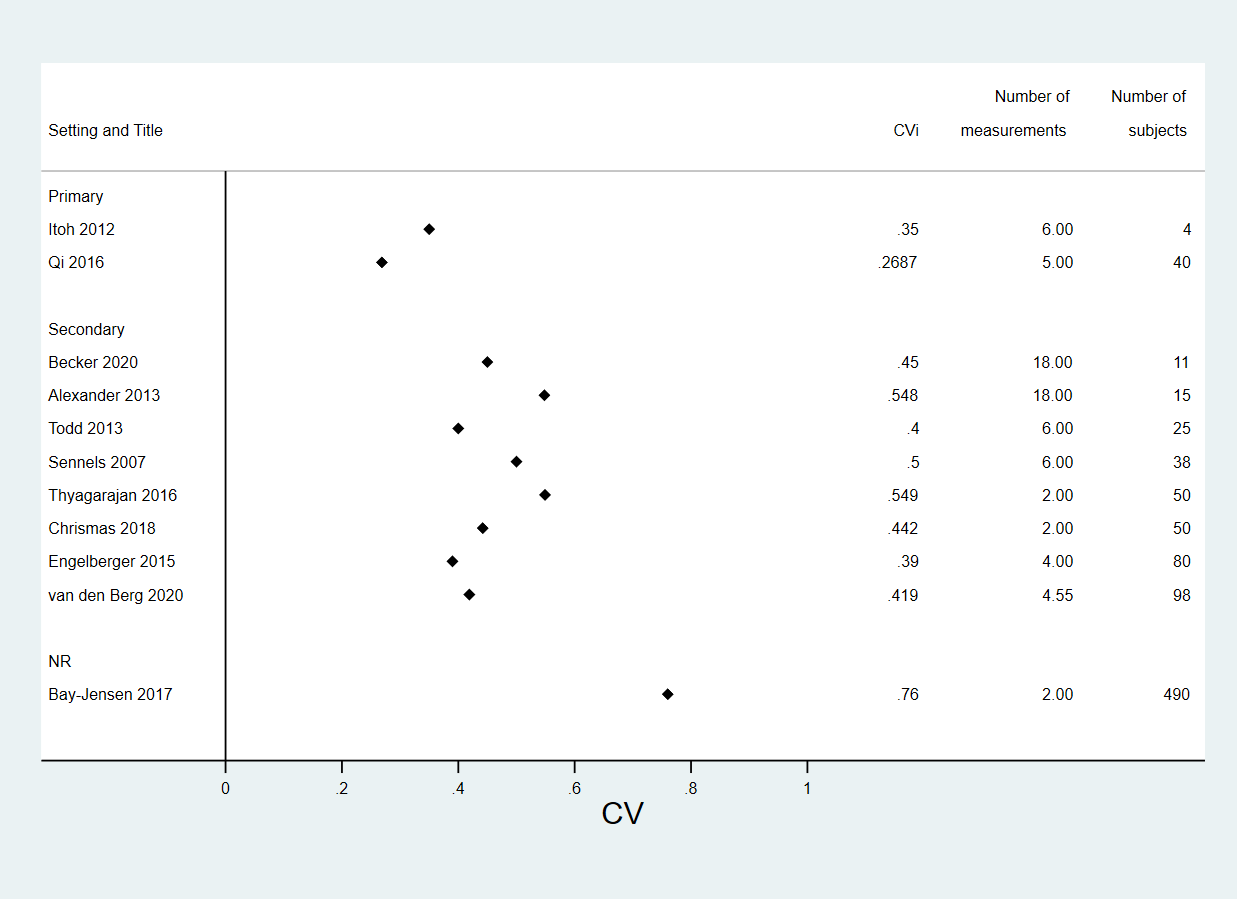


Fig 11 Forest plot of all papers that reported a CV for hsCRP, split by setting


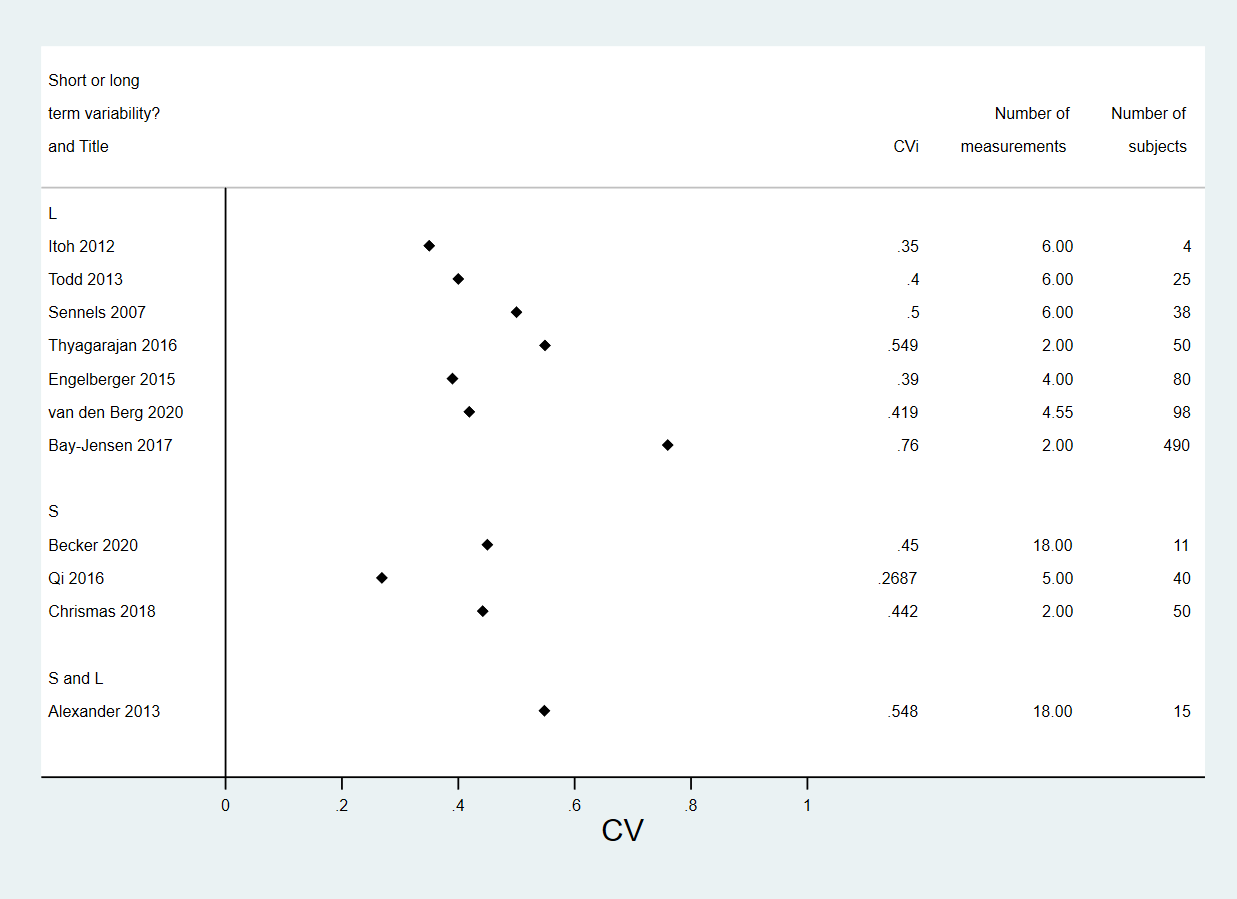


Fig 12 Forest plot of all papers that reported a CV for hsCRP, split by short (S) or long (L) term measurement of variability

### CRP ICCs


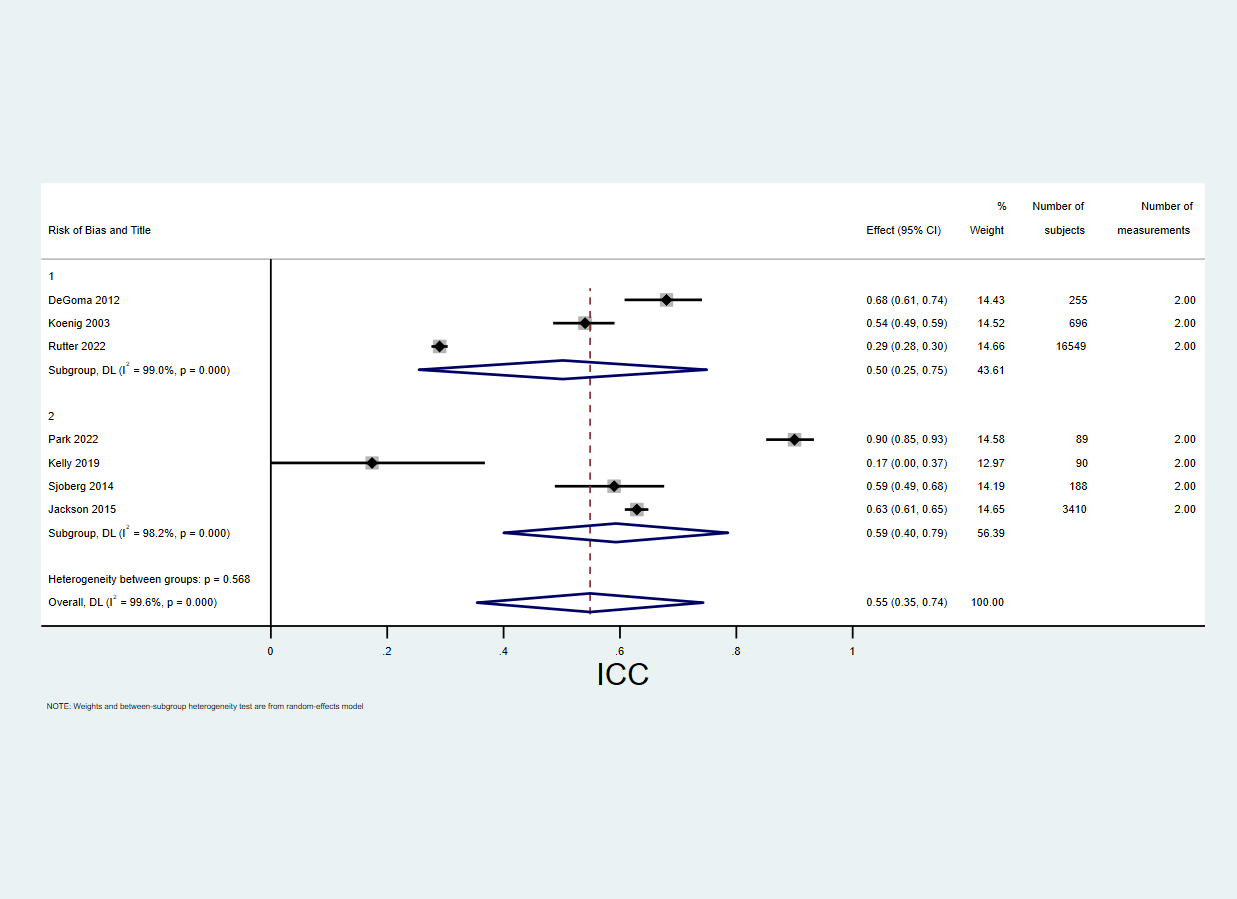


Fig 13 Forest plot of all papers that reported an ICC for CRP, split by Risk of Bias, with meta-analysis

### hsCRP ICCs


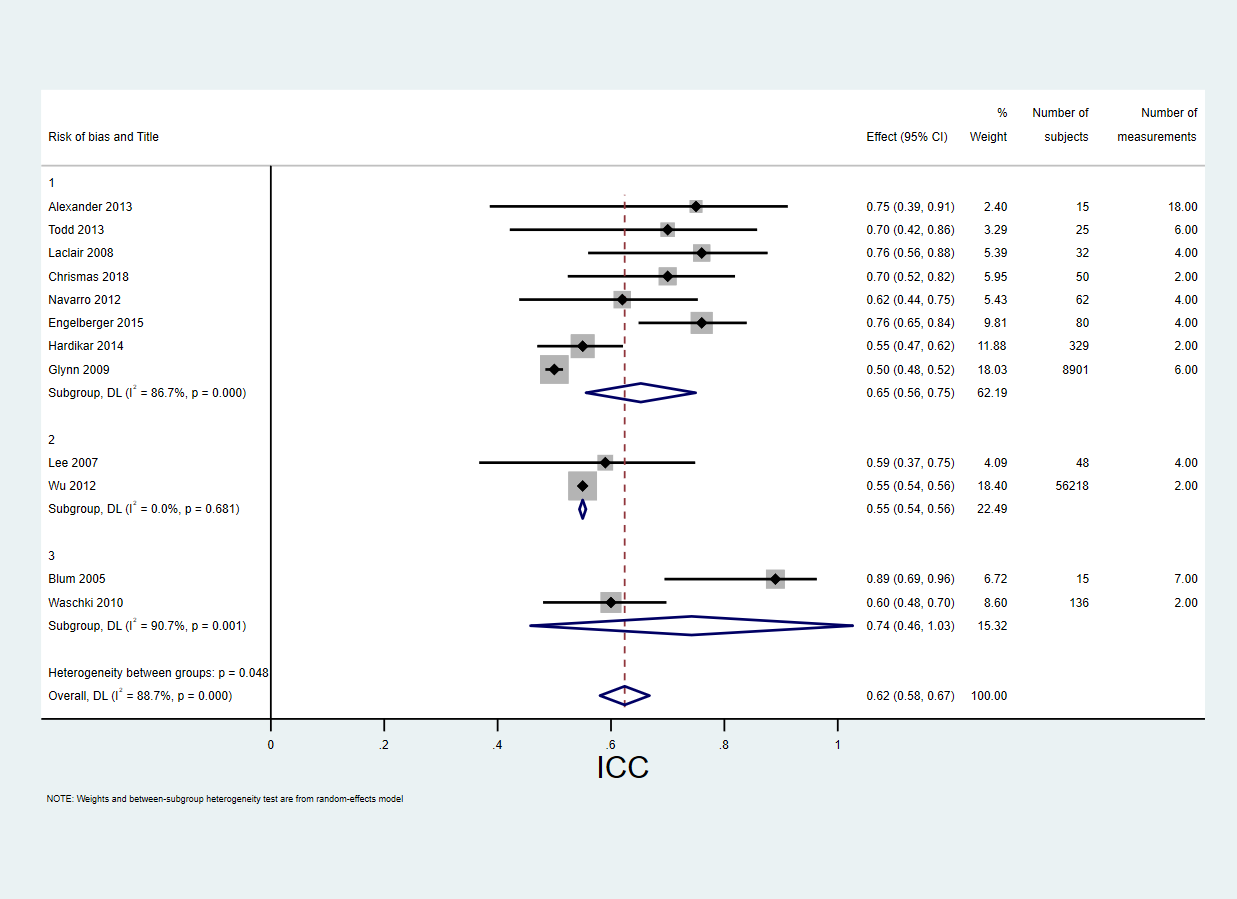


Fig 14 Forest plot of all papers that reported an ICC for hsCRP, split by Risk of Bias, with meta-analysis

# References

1. Nagy-Simon T, Hada A-M, Suarasan S, Potara M. Recent advances on the development of plasmon-assisted biosensors for detection of C-reactive protein. Journal of molecular structure. 2021;1246:131178.

2. Ansar W, Ghosh S. C-reactive protein and the biology of disease. Immunol Res. 2013;56(1):131-42.

3. Casas JP, Shah T, Hingorani AD, Danesh J, Pepys MB. C‐reactive protein and coronary heart disease: a critical review. J Intern Med. 2008;264(4):295-314.

4. Oppong R, Jit M, Smith RD, Butler CC, Melbye H, Mölstad S, et al. Cost-effectiveness of point-of-care C-reactive protein testing to inform antibiotic prescribing decisions. 2013.

5. Kaura A, Hartley A, Panoulas V, Glampson B, Shah ASV, Davies J, et al. Mortality risk prediction of high-sensitivity C-reactive protein in suspected acute coronary syndrome: A cohort study. PLOS Medicine. 2022;19(2):e1003911.

6. Nehring SM, Goyal A, Patel BC. C Reactive Protein. StatPearls. Treasure Island (FL): StatPearls Publishing

Copyright © 2022, StatPearls Publishing LLC.; 2022.

7. Pohanka M. Diagnoses Based on C-Reactive Protein Point-of-Care Tests. Biosensors (Basel). 2022;12(5):344.

8. Eccles S, Pincus C, Higgins B, Woodhead M. Diagnosis and management of community and hospital acquired pneumonia in adults: summary of NICE guidance. BMJ. 2014;349(dec03 4):g6722-g.

9. Ghazizadeh H, Kathryn Bohn M, Ghaffarian Zirak R, Kamel Khodabandeh A, Zare-Feyzabadi R, Saberi-Karimian M, et al. Comprehensive laboratory reference intervals for routine biochemical markers and pro-oxidant-antioxidant balance (PAB) in male adults. J Clin Lab Anal. 2020;34(11):e23470.

10. Cho SMJ, Lee H, Shim J-S, Kim HC. Sex-, Age-, and Metabolic Disorder-Dependent Distributions of Selected Inflammatory Biomarkers among Community-Dwelling Adults. Diabetes & metabolism journal. 2020;44(5):711-25.

11. Braga F, Panteghini M. Biologic variability of C-reactive protein: Is the available information reliable? Clin Chim Acta. 2012;413(15-16):1179-83.

12. Johnson TV, Abbasi A, Master VA. Systematic review of the evidence of a relationship between chronic psychosocial stress and C-reactive protein. Mol Diagn Ther. 2013;17(3):147-64.

13. Ain QU, Sarfraz M, Prasesti GK, Dewi TI, Kurniati NF. Confounders in Identification and Analysis of Inflammatory Biomarkers in Cardiovascular Diseases. Biomolecules. 2021;11(10).

14. Fraser Callum G. Biological Variation: From Principles to Practice. USA: AACC Press; 2001.

15. Hilderink JM, van der Linden N, Kimenai DM, Litjens EJR, Klinkenberg LJJ, Aref BM, et al. Biological Variation of Creatinine, Cystatin C, and eGFR over 24 Hours. Clin Chem. 2018;64(5):851-60.

16. Carlsen S, Petersen PH, Skeie S, Skadberg O, Sandberg S. Within-subject biological variation of glucose and HbA(1c) in healthy persons and in type 1 diabetes patients. Clinical Chemistry & Laboratory Medicine. 2011;49(9):1501-7.

17. EFLM. EFLM Biological Variation Database. [February 2022]. Available from: <https://biologicalvariation.eu/>.

18. Barak‐Corren Y, Barak‐Corren N, Gileles‐Hillel A, Heiman E. The effect of C‐reactive protein on chest X‐ray interpretation: A decision‐making experiment among pediatricians. Pediatr Pulmonol. 2021;56(6):1644-50. doi: 10.1002/ppul.25299.

19. Povoa P, Coelho L, Almeida E, Fernandes A, Mealha R, Moreira P, et al. Early identification of intensive care unit-acquired infections with daily monitoring of C-reactive protein: a prospective observational study. Critical Care (London, England). 2006;10(2):R63. PubMed PMID: 16635270.

20. Byron C. Wallace KS, Carla E. Brodley, Joseph Lau and Thomas A. Trikalinos, editor Deploying an interactive machine learning system in an evidence-based practice center: abstrackr. ACM International Health Informatics Symposium (IHI); 2012.

21. Mokkink LB, Boers M, van der Vleuten CPM, Bouter LM, Alonso J, Patrick DL, et al. COSMIN Risk of Bias tool to assess the quality of studies on reliability or measurement error of outcome measurement instruments: a Delphi study. BMC Med Res Methodol. 2020;20(1):293. Epub 2020/12/04. doi: 10.1186/s12874-020-01179-5. PubMed PMID: 33267819; PubMed Central PMCID: PMCPMC7712525.

22. Aarsand AK, Roraas T, Fernandez-Calle P, Ricos C, Diaz-Garzon J, Jonker N, et al. The Biological Variation Data Critical Appraisal Checklist: A Standard for Evaluating Studies on Biological Variation. Clin Chem. 2018;64(3):501-14. Epub 2017/12/10. doi: 10.1373/clinchem.2017.281808. PubMed PMID: 29222339.

23. Field AP. Is the meta-analysis of correlation coefficients accurate when population correlations vary? Psychol Methods. 2005;10(4):444-67. Epub 2006/01/06. doi: 10.1037/1082-989x.10.4.444. PubMed PMID: 16392999.

24. Kelly K, Mezuk B. Depression, Inflammation, and Metabolic Risk: A Genetically-Informed Exploratory Study. European Neuropsychopharmacology. 2019;29 (Supplement 3):S976-S7. PubMed PMID: 2001729225.

25. Alexander KS, Kazmierczak SC, Snyder CK, Oberdorf JA, Farrell DH. Prognostic utility of biochemical markers of cardiovascular risk: impact of biological variability. Clinical Chemistry & Laboratory Medicine. 2013;51(9):1875-82. PubMed PMID: 23648634.

26. Bailey D, Bevilacqua V, Colantonio DA, Pasic MD, Perumal N, Chan MK, et al. Pediatric within-day biological variation and quality specifications for 38 biochemical markers in the CALIPER cohort. Clin Chem. 2014;60(3):518-29. doi: 10.1373/clinchem.2013.214312.

27. Bay-Jensen AC, Bihlet A, Byrjalsen I, Andersen J, He Y, Siebuhr A, et al. Elevated levels of CRPM, an inflammatory biomarker correlating with disease activity in RA, are prognostic of radiographic knee OA. Osteoarthritis and Cartilage. 2017;25 (Supplement 1):S32. PubMed PMID: 620926908.

28. Becker M, Sperlich B, Zinner C, Achtzehn S. Intra-Individual and Seasonal Variation of Selected Biomarkers for Internal Load Monitoring in U-19 Soccer Players. Front Physiol. 2020;11:838. doi: <https://dx.doi.org/10.3389/fphys.2020.00838>. PubMed PMID: 32848822.

29. Block G, Dietrich M, Norkus E, Jensen C, Benowitz N, L., Morrow J, D. , et al. Intraindividual Variability of Plasma Antioxidants, Markers of Oxidative Stress, C-Reactive Protein, Cotinine, and Other Biomarkers. Epidemiology. 2006;17(4):404-12. doi: 10.1097/01.ede.0000220655.53323.e9.

30. Blum CA, Muller B, Huber P, Kraenzlin M, Schindler C, De Geyter C, et al. Low-grade inflammation and estimates of insulin resistance during the menstrual cycle in lean and overweight women. Journal of Clinical Endocrinology and Metabolism. 2005;90(6):3230-5. doi: <http://dx.doi.org/10.1210/jc.2005-0231>. PubMed PMID: 41014280.

31. Boenisch O, Ehmke KD, Heddergott A, Naoum C, Frei U, Schindler R. C-reactive-protein and cytokine plasma levels in hemodialysis patients. Journal of Nephrology. 2002;15(5):547-51. PubMed PMID: 12455722.

32. Bogaty P, Dagenais GR, Joseph L, Boyer L, Leblanc A, Belisle P, et al. Time variability of C-reactive protein: implications for clinical risk stratification. PLoS ONE [Electronic Resource]. 2013;8(4):e60759. PubMed PMID: 23579782.

33. Bogaty P, Brophy JM, Boyer L, Simard S, Joseph L, Bertrand F, et al. Fluctuating Inflammatory Markers in Patients With Stable Ischemic Heart Disease. Arch Intern Med. 2005;165(2):221-6. doi: 10.1001/archinte.165.2.221.

34. Browning LM, Jebb SA, Mishra GD, Cooke JH, O'Connell MA, Crook MA, et al. Elevated sialic acid, but not CRP, predicts features of the metabolic syndrome independently of BMI in women. International Journal of Obesity & Related Metabolic Disorders: Journal of the International Association for the Study of Obesity. 2004;28(8):1004-10. PubMed PMID: 15211367.

35. Carobene A, Aarsand AK, Guerra E, Bartlett WA, Coskun A, Diaz-Garzon J, et al. European Biological Variation Study (EuBIVAS): Within- and Between-Subject Biological Variation Data for 15 Frequently Measured Proteins. Clinical Chemistry. 2019;65(8):1031-41. doi: <https://dx.doi.org/10.1373/clinchem.2019.304618>. PubMed PMID: 31171528.

36. Cho LW, Jayagopal V, Kilpatrick ES, Atkin SL. The biological variation of C-reactive protein in polycystic ovarian syndrome. Clinical Chemistry. 2005;51(10):1905-7. PubMed PMID: 16189386.

37. Chrismas B, Taylor L, Smith A, Pemberton P, Siegler JC, Midgley AW. Reproducibility of measurement techniques used for creatine kinase, interleukin-6 and high-sensitivity C-reactive protein determination over a 48 h period in males and females. Measurement in Physical Education and Exercise Science. 2018;22(3):191-9. PubMed PMID: 619735473.

38. Clark GH, Fraser CG. Biological variation of acute phase proteins. Annals of Clinical Biochemistry. 1993;30(Pt 4):373-6. PubMed PMID: 7691039.

39. Corte Z, Venta R. Biological variation of metabolic cardiovascular risk factors in haemodialysis patients and healthy individuals. Annals of Translational Medicine. 2020;8(6). doi: <http://dx.doi.org/10.21037/atm.2020.03.26>. PubMed PMID: 631447901.

40. de Maat MPM, de Bart ACW, Hennis BC, Meijer P, Havelaar AC, Mulder PGH, et al. Interindividual and Intraindividual Variability in Plasma Fibrinogen, TPA Antigen, PAI Activity, and CRP in Healthy, Young Volunteers and Patients With Angina Pectoris. Arterioscler Thromb Vasc Biol. 1996;16(9):1156-62. doi: 10.1161/01.ATV.16.9.1156.

41. DeGoma EM, French B, Dunbar RL, Allison MA, Mohler ER, 3rd, Budoff MJ. Intraindividual variability of C-reactive protein: the Multi-Ethnic Study of Atherosclerosis. Atherosclerosis. 2012;224(1):274-9. PubMed PMID: 22846611.

42. d'Eril GM, Anesi A, Maggiore M, Leoni V. Biological variation of serum amyloid A in healthy subjects. Clinical Chemistry. 2001;47(8):1498-9. PubMed PMID: 11468251.

43. Engelberger RP, Limacher A, Kucher N, Baumann F, Silbernagel G, Benghozi R, et al. Biological variation of established and novel biomarkers for atherosclerosis: Results from a prospective, parallel-group cohort study. Clinica Chimica Acta. 2015;447:16-22. PubMed PMID: 25979692.

44. Franzini C, Scapallato, L., Vanoni, G. Variabilita biologica ed analitica della anti-streptolisina O e di proteine della fase acuta del siero. Biochimica Clinica. 1993;17:315-9.

45. Gasco J, Inigo V, Mascaros V, Bernabeu R, Servera M. Biological variation of beta2-microglobulin in haemodiafiltration patients. NDT Plus. 2010;3:iii459. PubMed PMID: 70484668.

46. Glynn RJ, MacFadyen JG, Ridker PM. Tracking of high-sensitivity C-reactive protein after an initially elevated concentration: The JUPITER study. Clinical Chemistry. 2009;55(2):305-12. doi: <http://dx.doi.org/10.1373/clinchem.2008.120642>. PubMed PMID: 354152794.

47. Guy JH, Edwards AM, Miller CM, Deakin GB, Pyne DB. Short-term reliability of inflammatory mediators and response to exercise in the heat. Journal of sports sciences. 2017;35(16):1622-8. doi: <http://dx.doi.org/10.1080/02640414.2016.1227464>. PubMed PMID: 618863746.

48. Hardikar S, Song X, Kratz M, Anderson GL, Blount PL, Reid BJ, et al. Intraindividual variability over time in plasma biomarkers of inflammation and effects of long-term storage. Cancer Causes and Control. 2014;25(8):969-76. doi: <http://dx.doi.org/10.1007/s10552-014-0396-0>. PubMed PMID: 53160337.

49. Hosogaya S, Naito K, Sakamoto M, Osada M, Yatomi Y, Ozaki Y. Biological inter- and intra-individual variations of serum immunochemical constituents and their allowable limits of analytical error. [Japanese]. Rinsho byori. 1999;The Japanese journal of clinical pathology. 47(9):875-80. PubMed PMID: 129471599.

50. Itoh H, Mori I, Matsumoto Y, Maki S, Ogawa Y. Seasonal and inter-day variation in serum high-sensitivity C-reactive protein in Japanese male workers: A longitudinal study. Industrial Health. 2012;50(1):60-3. PubMed PMID: 364261684.

51. Jackson SE, van Jaarsveld CH, Beeken RJ, Gunter MJ, Steptoe A, Wardle J. Four-year stability of anthropometric and cardio-metabolic parameters in a prospective cohort of older adults. Biomarkers in Medicine. 2015;9(2):109-22. doi: <https://dx.doi.org/10.2217/bmm.14.78>. PubMed PMID: 25689899.

52. Jain P, Kadeangadi DM. Depression and anxiety among adult patients with type II diabetes mellitus- a descriptive study in Urban North Karnataka. Journal of Clinical and Diagnostic Research. 2020;14(6):LC11-LC4. doi: <http://dx.doi.org/10.7860/JCDR/2020/44048.13793>. PubMed PMID: 2006132069.

53. Jung D, Jang J, Kwong E, Song J, Quon B. Serum CRP and calprotectin to diagnose CF pulmonary exacerbations. Pediatr Pulmonol. 2019;54 (Supplement 2):353.

54. Koenig W, Sund M, Frohlich M, Lowel H, Hutchinson WL, Pepys MB. Refinement of the association of serum C-reactive protein concentration and coronary heart disease risk by correction for within-subject variation over time: The MONICA Augsburg studies, 1984 and 1987. American Journal of Epidemiology. 2003;158(4):357-64. doi: <http://dx.doi.org/10.1093/aje/kwg135>. PubMed PMID: 41288803.

55. Kolsum U, Roy K, Starkey C, Borrill Z, Truman N, Vestbo J, et al. The repeatability of interleukin-6, tumor necrosis factor-alpha, and C-reactive protein in COPD patients over one year. International Journal of Copd. 2009;4:149-56. PubMed PMID: 19436686.

56. Laclair R, O'Neal K, Ofner S, Sosa MJ, Labarrere CA, Moe SM. Precision of biomarkers to define chronic inflammation in CKD. American Journal of Nephrology. 2008;28(5):808-12. doi: <http://dx.doi.org/10.1159/000135692>. PubMed PMID: 352289316.

57. Lee SA, Kallianpur A, Xiang YB, Wen W, Cai Q, Liu D, et al. Intra-individual variation of plasma adipokine levels and utility of single measurement of these biomarkers in population-based studies. Cancer Epidemiology Biomarkers and Prevention. 2007;16(11):2464-70. doi: <http://dx.doi.org/10.1158/1055-9965.EPI-07-0374>. PubMed PMID: 351196382.

58. Macy EM, Hayes TE, Tracy RP. Variability in the measurement of C-reactive protein in healthy subjects: implications for reference intervals and epidemiological applications. Clinical Chemistry. 1997;43(1):52-8. PubMed PMID: 8990222.

59. Malaponte G, Libra M, Bevelacqua Y, Merito P, Fatuzzo P, Rapisarda F, et al. Inflammatory status in patients with chronic renal failure: the role of PTX3 and pro-inflammatory cytokines. International Journal of Molecular Medicine. 2007;20(4):471-81. PubMed PMID: 17786277.

60. Meyer T, Meister S. Routine blood parameters in elite soccer players. International Journal of Sports Medicine. 2011;32(11):875-81. PubMed PMID: 22020850.

61. Nasermoaddeli A, Sekine M, Kagamimori S. Intra-individual variability of high-sensitivity C-reactive protein: age-related variations over time in Japanese subjects. Circulation Journal. 2006;70(5):559-63. PubMed PMID: 16636490.

62. Navarro SL, Brasky TM, Schwarz Y, Song X, Wang CY, Kristal AR, et al. Reliability of serum biomarkers of inflammation from repeated measures in healthy individuals. Cancer Epidemiology Biomarkers and Prevention. 2012;21(7):1167-70. doi: <http://dx.doi.org/10.1158/1055-9965.EPI-12-0110>. PubMed PMID: 365192757.

63. Nunes LA, Brenzikofer R, de Macedo DV. Reference change values of blood analytes from physically active subjects. European Journal of Applied Physiology. 2010;110(1):191-8. PubMed PMID: 20446091.

64. Ockene IS, Matthews CE, Rifai N, Ridker PM, Reed G, Stanek E. Variability and Classification Accuracy of Serial High-Sensitivity C-Reactive Protein Measurements in Healthy Adults. Clin Chem. 2001;47(3):444-50. doi: 10.1093/clinchem/47.3.444.

65. Park S, Phillips JE, Saiphoklang N, Wilgus ML, Buhr RG, Cooper CB, et al. Stability of Commonly Evaluated Biomarkers in Clinically Stable COPD. American Journal of Respiratory and Critical Care Medicine Conference: International Conferenceof the American Thoracic Society, ATS. 2022;205(1). doi: <https://dx.doi.org/10.1164/ajrccm-conference.2022.205.1_MeetingAbstracts.A1095>. PubMed PMID: 638408055.

66. Platz EA, Sutcliffe S, De Marzo AM, Drake CG, Rifai N, Hsing AW, et al. Intra-individual variation in serum C-reactive protein over 4 years: an implication for epidemiologic studies. Cancer Causes & Control. 2010;21(6):847-51. PubMed PMID: 20135215.

67. Qi Z, Chen Y, Zhang L, Ma X, Wang F, Cheng Q, et al. Biological variations of thirteen plasma biochemical indicators. Clinica Chimica Acta. 2016;452:87-91. PubMed PMID: 26561925.

68. Riese H, Vrijkotte TG, Meijer P, Kluft C, de Geus EJ. Diagnostic strategies for C-reactive protein. BMC Cardiovascular Disorders. 2002;2:9. PubMed PMID: 12049676.

69. Rubin LH, Benning L, Keating SM, Norris PJ, Burke-Miller J, Savarese A, et al. Variability in C-reactive protein is associated with cognitive impairment in women living with and without HIV: a longitudinal study. Journal of Neurovirology. 2018;24(1):41-51. PubMed PMID: 29063513.

70. Rudez G, Meijer P, Spronk HM, Leebeek FW, ten Cate H, Kluft C, et al. Biological variation in inflammatory and hemostatic markers. Journal of Thrombosis & Haemostasis. 2009;7(8):1247-55. PubMed PMID: 19566543.

71. Rutter CE, Millard LAC, Borges MC, Lawlor DA. Exploring regression dilution bias using repeat measurements of 2858 variables in up to 49 000 UK Biobank participants. medRxiv. 2022;15. doi: <https://dx.doi.org/10.1101/2022.07.13.22277605>. PubMed PMID: 2019603053.

72. Sakkinen PA, Macy EM, Callas PW, Cornell ES, Hayes TE, Kuller LH, et al. Analytical and biologic variability in measures of hemostasis, fibrinolysis, and inflammation: assessment and implications for epidemiology. American Journal of Epidemiology. 1999;149(3):261-7. PubMed PMID: 9927222.

73. Sennels HP, Jacobsen S, Jensen T, Hansen MS, Ostergaard M, Nielsen HJ, et al. Biological variation and reference intervals for circulating osteopontin, osteoprotegerin, total soluble receptor activator of nuclear factor kappa B ligand and high-sensitivity C-reactive protein. Scandinavian Journal of Clinical & Laboratory Investigation. 2007;67(8):821-35. PubMed PMID: 17852826.

74. Shaw N, White H, Denman S, Peckham D. The impact of disease severity and clinical variation on self-reported adherence. Journal of Cystic Fibrosis. 2014;13:S117.

75. Sjoberg B, Snaedal S, Stenvinkel P, Qureshi AR, Heimburger O, Barany P. Three-month variation of plasma pentraxin 3 compared with C-reactive protein, albumin and homocysteine levels in haemodialysis patients. Clinical Kidney Journal. 2014;7(4):373-9. doi: <http://dx.doi.org/10.1093/ckj/sfu071>. PubMed PMID: 373660950.

76. Thyagarajan B, Howard AG, Durazo-Arvizu R, Eckfeldt JH, Gellman MD, Kim RS, et al. Analytical and biological variability in biomarker measurement in the Hispanic Community Health Study/Study of Latinos. Clinica Chimica Acta. 2016;463:129-37. PubMed PMID: 27756543.

77. Thompson AE, Pope JE. The erratic C-reactive protein: a novel outcome measure for longitudinal disease activity in rheumatoid arthritis. Clinical and Experimental Rheumatology. 2022;40(7):1411-6. doi: <https://dx.doi.org/10.55563/clinexprheumatol/tdp7to>. PubMed PMID: 2019308754.

78. Todd J, Simpson P, Estis J, Torres V, Wub AH. Reference range and short- and long-term biological variation of interleukin (IL)-6, IL-17A and tissue necrosis factor-alpha using high sensitivity assays. Cytokine. 2013;64(3):660-5. PubMed PMID: 24128872.

79. Tsirpanlis G, Bagos P, Ioannou D, Bleta A, Marinou I, Lagouranis A, et al. The variability and accurate assessment of microinflammation in haemodialysis patients. Nephrology Dialysis Transplantation. 2004;19(1):150-7. PubMed PMID: 14671050.

80. van den Berg VJ, Umans VAWM, Brankovic M, Oemrawsingh RM, Asselbergs FW, van der Harst P, et al. Stabilization patterns and variability of hs-CRP, NT-proBNP and ST2 during 1 year after acute coronary syndrome admission: results of the BIOMArCS study. Clinical chemistry and laboratory medicine. 2020;08. PubMed PMID: 631704136.

81. Waschki B, Schaper M, Sack A, Paasch K, Mueller K, Feindt B, et al. Cardiovascular biomarkers in COPD patients during three years of follow-up. American Journal of Respiratory and Critical Care Medicine Conference: American Thoracic Society International Conference, ATS. 2010;181(1 MeetingAbstracts).

82. White H, Shaw N, Denman S, Pollard K, Wynne S, Peckham DG. Variation in lung function as a marker of adherence to oral and inhaled medication in cystic fibrosis. European Respiratory Journal. 2017;49(3):03. PubMed PMID: 28275171.

83. Wu S, Li Y, Jin C, Yang P, Li D, Li H, et al. Intra-individual variability of high-sensitivity C-reactive protein in Chinese general population. International Journal of Cardiology. 2012;157(1):75-9. PubMed PMID: 21215477.
