# Supplementary material for: Within-subject variation of C-reactive protein and high-sensitivity C-reactive protein: A systematic review and meta-analysis
Source: PLoS One. 2024 Nov 1;19(11):e0304961. doi: 10.1371/journal.pone.0304961 (PMC11530069; doi:10.1371/journal.pone.0304961)
Supplement: Attachment — Submitted filename: rebuttal letter July 23 v2.docx [file pone.0304961.s003.docx]

Dear Dr Chenette,

Re: PONE-D-23-07133, “*Within-subject Variation of C-reactive protein and high-sensitivity C-reactive protein: A systematic review and meta-analysis”,* by Gough et al.

Thank you for giving us the opportunity to resubmit our paper. As mentioned in my previous query letter, we feel there was a misunderstanding regarding our reasons for exclusion of studies at the title and abstract and the full text screening stages. Most importantly, where we stated that most papers were excluded for lack of a recognised measure of variability, this should have been more clearly stated that most papers were excluded for lack of any reported data on variability. Also of importance is that because the initial searches for this paper were combined with a similar paper concerning HbA1c, studies that only reported variability for HbA1c and not CRP were excluded at the full text stage. This has been clarified in the revised manuscript at lines 210-220.

The specific reviewer’s comments are addressed below

The reviewer commented:

“1. Study selection: The study initially retrieved a large number of non-duplicate studies (2675), but only a small percentage (13.3%) were included after title and abstract screening. It would be helpful to provide more information on the specific criteria used for study selection and the reasons for excluding the majority of studies based on title and abstract.”

“2. Full text screening: After full text screening, 83.1% of the screened studies were excluded. The main reason for exclusion was the lack of reporting of a recognized measure of variability. This indicates a potential limitation in the literature, but it would be useful to elaborate on the specific measures of variability that were required and why their absence led to exclusion.”

We have clarified that 2320 studies (86.7%) were excluded after title and abstract screening because it was clear they did not include information on variability of either HbA1c or CRP. We also clarified that after full text screening, 295 more studies were excluded mainly because they did not report any variability data or they reported variability for HbA1c only (not CRP). Some were excluded because they were reviews and did not report primary data, they reported duplicate data or they reported a non-standard measure of variability. This is described in detail in lines 210-220 and in Figure 1

“3. Variability measures: The study reports the coefficient of variation (CV) and intraclass correlation coefficient (ICC) as measures of variability. While the median and range of these measures are presented, it would be helpful to discuss the clinical significance of these values. Are there established thresholds for what is considered low or high variability in CRP and hsCRP levels?”

We are not aware of any standard thresholds for what is considered low or high biological variability, and the definition of low or high variability would vary by individual parameter and the way in which it is used to inform clinical decision-making. See lines 318-325. In fact this systematic review forms part of a larger body of work which investigates the implications of variability of a number of clinical parameters for clinical decision-making.

There appears to be no point 4.

5. Publication bias: The study mentions that risk of bias due to missing results was deemed to be low because the outcome measure of the review was not the primary outcome of the individual studies. However, it is important to acknowledge that the exclusion of studies with missing results may introduce bias if the missing data are systematically related to the variability measures. Further discussion on the potential impact of publication bias on the findings would be valuable.”

As has been clarified, most studies were excluded because they did not report any variability measures. However, the reviewers point about the potential for bias has been included, see lines 279-281.

Areas of improvement:

“1. Clarity in reporting: The study would benefit from clearer and more concise reporting. Some sentences are overly long and contain multiple pieces of information, making it challenging to follow the flow of the text. Breaking down the information into separate sentences or paragraphs would enhance readability.”

The manuscript has been revised for readability.

“2. Practical implications: It would be helpful to discuss the practical implications of the findings. For example, how do the reported variability measures impact the interpretation of CRP and hsCRP levels in clinical practice? Are there any implications for the use of these biomarkers in diagnostic or monitoring settings?”

This has been addressed on lines 325-322. In brief, the implications of within-individual variation depend both on the degree of within-individual variation of the parameter and the disparity in measured values between healthy and diseased populations.

3. “Future research directions: Given the findings and limitations of the included studies, it would be valuable to suggest potential areas of improvement and future research directions. This could include recommendations for standardizing the reporting of variability measures or identifying specific populations or conditions where more research is needed.”

This has been addressed on lines 352-360 where we make recommendations for reporting variability, the need for estimates of variability in diseased populations and the need to explore the implications of within-individual variation for clinical guidelines and for clinical decision making.

Thank you for your time and once again apologies for our previous lack of clarity.

Yours sincerely,

Alex Gough MA VetMB MRCVS

Institute of Applied Health Research

University of Birmingham
