# Supplementary material for: Within-subject variation of C-reactive protein and high-sensitivity C-reactive protein: A systematic review and meta-analysis
Source: PLoS One. 2024 Nov 1;19(11):e0304961. doi: 10.1371/journal.pone.0304961 (PMC11530069; doi:10.1371/journal.pone.0304961)
Supplement: Attachment — Submitted filename: Response to reviewers.docx [file pone.0304961.s004.docx]

Dear Dr Chenette,

Re: PONE-D-23-37309 “Within-subject variation of C-reactive protein and high-sensitivity C-reactive protein: A systematic review and meta-analysis,” by Gough et al.

Thank you for giving us the opportunity to revise our paper. A revised manuscript is enclosed, and a detailed response to the reviewers follows below.

Yours sincerely,

Alex Gough MA VetMB MRCVS

Institute of Applied Health Research

University of Birmingham

Reviewer #1: Review

Authors performed a meta-analysis of studies reporting variability in different populations. They report large intraindidivual variability over time. While this is interesting to clinicians (potential overuse of CRP), there are major concerns regarding the design of the meta-analysis and the quality of the underlying studies.

Thank you for the comment. One of the aims of the systematic review is to identify studies of intra-individual variability and characterise their quality.

Abstract
Line 28-29: A systematic review and meta-analysis was performed to estimate within-subject variability of CRP and hsCRP. ◊ Please add, that variability over time was measured and state which time period was considered.

We have clarified that by within-individual variability we mean variability over time within the same individual. There were no time restrictions on the interval between measurements within the same individual. Line 28

Introduction

Line 70: while it may be true that hsCRP can predict the risk of cardiovascular disease I do not agree that this it is used for this purpose in the clinic often. I would say that the most important use of hsCRP is to identify patients with known atherosclerotic cardiovascular disease with residual inflammatory risk.

Line 95: I do not understand the sentence – “duetocause”?

Thank you for this comment. We have revised this sentence.

Line 118-121: What are the authors trying to say? Please rephrase.

Thank you for this comment. We have revised this sentence.

Methods:
Line 137-142: I would suggest a statistician to check the methodology as I myself am not an expert in statistics.

Alice Sitch (co-author) is a statistician.

Line 143-144: “There was no restriction on time of publication, language of publication, population, setting or sample size”. Overall, this is problematic. This design limits the validity as biological variability will differ between populations and larger sample sizes are less likely to report random results. For example, including a study with 4 patients in the analysis is questionable.

We agree that variability will differ between populations and that random error may be smaller in larger studies. However no previous systematic review or meta-analysis of within-individual variability in CRP of this size or methodology has been carried out. We therefore first intended to identify all available studies and then comment on differences between studies.

What were the intervals between CRP measurements?

See response above. All intervals were allowed.

Were the studies included conducted specifically to assess CRP/hsCRP variability? If not, how can they ensure that intraindividual variability was measured and not treatment effects or disease states (trauma, infection, cancer etc.) were measured? The authors need to explain how the results they report are meaningful rather than statistical “noise”.

Already mentioned in lines 145-147. Few studies were conducted specifically to assess within-individual variability, but the purpose of this review was to include as many studies as possible that reported within-individual variability. Studies were excluded where there was an intervention between measurements or an unstable disease state.

Line 154: Foreign language papers were translated by Google translate software. – this is problematic as it can introduce mistakes.

Translation software was used mainly to identify whether a paper was eligible for inclusion. The only foreign language paper included was Franzini, and the figures in this are also included in a table in an English language review by Braga and Panteghini ( DOI: 10.1016/j.cca.2012.04.010)

Results:

Regarding Line 222-223: CRP values will naturally vary over time, particularly if the study features clinical trials, as interventions will affect CRP levels (e.g. statin treatment). Later the authors state that CRP levels measured before and after interventions led to exclusion of the study. There were six clinical trials included (placebo). However, even the placebo may theoretically affect CRP levels (patient may behave differently if he believes being treated). I do not believe these studies should be included. I believe answering this question requires more controlled conditions and a prospective cohort study design.

Studies with measurements taken before and after an intervention are excluded. We are not aware of any evidence that placebo affects within-individual variability, and we have mentioned this in lines 353-355.

Line 329: This is particularly true when single number cut-off points are used, for example “do not use antibiotics if CRP <20mg/L”, since a measured value of for example 18mg/L may represent a much higher (or much lower) true mean for that individual. Clinicians should be aware that a single measurement, or even multiple measurements, may not be an accurate reflection of the true mean CRP for an individual patient. – From a clinician’s perspective this statement is not required. Nobody would start antibiotics based alone on CRP. Please rephrase. In fact, many clinicians do not use CRP at all when assessing need for CRP. I believe that many factors influence CRP increase which is the more significant clinical problem, rather than a individual variability. This should be commented on in the discussion.

Individual variability may however be more important regarding hsCRP levels, particularly in the setting of cardiovascular disease. Authors should discuss this.

These points are addressed in lines 332-338

**Reviewer #2: At first I would like to thank the editor for the opportunity to review the paper of Gough et al.**

1.) Impact. The authors should outline the added value of this study, especially compared to the existing, updated meta-analyses of the EFLM database, including different health conditions. What additional knowledge do the authors' results provide and how should they be interpreted in comparison to the results of the existing meta-analysis?

Our search strategy is more consistent and transparent than that of the EFLM database, and includes a larger number of studies. Lines 298-304

2.) Readability and Typography. The study would profit from breaking down the main information in shorter sentences. Authors should as well remain consistent in the way they write out numbers or describe them as digits. Some parts of the study should be proofread for easier understanding (for example lines 46, 95…)

Lines 46 and 95 corrected, manuscript proof read and sentences shortened.

3.) Consistency in reporting results. The study would improve if the results were pointed out clearer.
The authors should clarify the following:
a) Report of all possible scores (1-4) of “risk of bias” in table 3.

Table 3 revised.

b) The authors report “In 22 (36.7%) studies the within-subject biological variation (CVI) was reported, while…”. Opposing this, about 32 observations of CV are mentioned later on, as well as in Figure 2 and 3.

It would be helpful to point out how many studies in which group (CRP vs hsCRP) were reporting CVI, CVT or CV.

Information added in lines 235-248
